# Compound Dislocation

**Published:** 1882-06

**Authors:** C. C. F. Gay


### Clinical Reports.

### Compound Dislocation.

By Dr. C. C. F. Gay.

Compound dislocations of the large joints are regarded as the most serious injury that a limb can be subjected to; more serious, in fact, than compound fractures. Primary amputation or resection is not unfrequently required, especially when the lower extremities are the seat of injury. Amputation is a surgical resource to be employed where there is extensive laceration of the soft parts or injury to blood vessels. The same rules of treatment for the lower, cannot be applied to the upper extremities. It will rarely be found necessary to amputate for compound dislocation of the upper extremities; resection is an alternative which the surgeon will chose rather than amputation. The following in an illustrative case of conservative treatment.

### COMPOUND DISLOCATION OF THE RIGHT TIBIA-ASTRAGALOID ARTICULATION. REDUCTION; RECOVERY WITH SCARCELY ANY DEFORMITY OR IMPAIRMENT OF MOTION.

J. S., æt. 38 years, on April 30th, 1881, was driving, when the horse, which was atttached to a high-seated lumber wagon, took fright and ran away. The driver was thrown from the vehicle, striking violently upon the pavement. The accident occurred at 11 A. M.; I saw the patient, at the request of Dr. Van Peyma, at 2 P. M. The foot was abducted; toes everted; the extremities of the tibia and internal malleolus protruded two inches through the integument, exposing the articulating surface of the tibia; external malleolus was fractured; there was some hemorrhage. Patient was etherized. I applied an extending band around the foot and heel, by means of which reduction was readily effected without enlarging the wound; strong traction was required. The upper margin of skin was folded inward upon itself which was restored by my forefinger.

The limb was secured to a wide and well padded splint, and warm water dressings applied. The patient did well.

In seven weeks he called at my office. He used crutches.

A note made at that time in my record book, says: Wound healed; foot somewhat abducted and toe everted; motion in the joint; bears some weight upon the foot.

Five months after date of injury the ankle shows but little deformity, and there is good motion of the joint and use of the limb. It should be added that there was also axillary luxation of the right shoulder, which was at once reduced by Dr. Van Peyma.

COMPOUND DISLOCATION AT THE RADIO-CARPAL ARTICULATION. RESECTION; RECOVERY WITH PROMISE OF A USEFUL HAND.

J. H., æt. 16 years, fell, in a planing mill, on January 30th, 1882, and sustained a compound dislocation of the left radius. Dr. Van Peyma, who had the patient in charge, called me in consultation on Feb. 20th, or three weeks after the injury occurred.

The lad was considerably emaciated and had lost much blood. The hand was much swollen and abscesses had formed in it; extremity of radius was exposed; was of a dark color; roughened, denuded of periosteum and becoming carious.

Three ligatures had been the evening before applied around the radial artery through the tissues, but the circulation was maintained in the hand by the ulner artery. Patient was placed upon the table and etherized. I first removed the ligatures—after applying the tourniquet to the brachial artery—then laid open the wound and resected an inch and a quarter of the radius by the chain saw. There was considerable venous hemorrhage; the boy very pale, pulse feeble and rapid. Hypodermic injections of brandy were resorted to. There was no displacement or exposure of the extremity of the ulna. The hand was freely incised upon its palmer aspect and drainage tube directed to be used. Sponge was placed over the wound with compress and bandage, and the patient placed in bed with his hand and arm resting upon a pillow; after a little rest in bed the patient felt quite comfortable.

March 2d, twelve days after the operation, the wound was filling up with healthy granulations; swelling of the hand was subsiding; the boy had a good appetite and is improving in all respects. On April 24th I received a call from the boy, who came to show his hand, and he is much elated over the result. Recovery seems complete with promise in a few months of a useful hand with movable wrist joint.

### COMPOUND DISLOCATION OF LEFT ELBOW; AMPUTATION; RECOVERY.

A. K., æt. 12 years, got his arm caught in belting of a coffee and spice mill and was drawn up to the ceiling, causing a dislocation at the elbow joint. The humerus up to three or four inches of the shoulder was exposed and denuded of flesh. Soft parts were much lacerated and the brachial artery severed; pulsation felt in it down nearly to the elbow; no pulsation in forearm. Patient was etherized. Assisted by Dr. O'Brien, who brought the patient to me, I amputated by anterior and posterior flaps close to the shoulder joint; five vessels required ligation. The following day the pulse was 130, temperature 103; from this time forward the patient progressed rapidly to recovery.

